# Donor Derived Cell Free DNA in Kidney Transplantation: The Circa 2020–2021 Update

**DOI:** 10.3389/ti.2022.10448

**Published:** 2022-06-01

**Authors:** Sam Kant, Daniel C. Brennan

**Affiliations:** ^1^ Division of Nephrology, Department of Medicine, School of Medicine, Johns Hopkins University, Baltimore, MD, United States; ^2^ Comprehensive Transplant Center, Johns Hopkins University School of Medicine, Baltimore, MD, United States

**Keywords:** kidney, biomarker, rejection, transplant, cell free DNA

## Abstract

The routine surveillance of kidney transplant allografts has relied on imperfect non-invasive biomarkers such as creatinine and urinary indices, while the gold standard allograft biopsy is associated with risk of bleeding, organ injury and sampling errors. Donor derived cell free DNA (dd-cfDNA) is being employed as a biomarker that addresses limitations of these surveillance methods, albeit has inherent drawbacks. This review provides an update on the enhanced understanding of dd-cfDNA and its expanded use beyond the conventional indication of detecting allograft rejection.

## Introduction

In the past 5 decades of the successful practice of kidney transplantation, a biomarker for monitoring of allograft rejection continued to elude the field. Donor derived cell free DNA (dd-cfDNA) has gained widespread utility as that biomarker in the transplant community since its introduction. After the initial demonstration of its use in detecting T-cell mediated and antibody mediated rejection in kidney transplantation (1), multiple studies have looked to further validate it and address challenges in diagnosis and interpretation. In addition, the application of dd-cfDNA is starting to expand beyond the conventional use of rejection. This includes monitoring of the effect of non-HLA antibodies, oncologic therapy, and opportunistic infections (2-4). The objective of this review is to provide an update on these newly elucidated facets of dd-cfDNA.

## Adding Nuance to the Biomarker

A multitude of clinical studies have documented the efficacy of dd-cfDNA in detecting rejection, however, is role in surveillance of kidney allografts in routine clinical practice has not been well elucidated. The ADMIRAL study (Assessing AlloSure Dd-cfDNA, Monitoring Insights of Renal Allografts with Longitudinal Surveillance; NCT04566055) looked to address this aspect through a large, multicenter, observational cohort study of kidney transplant recipients monitored with dd-cfDNA for ≤3 years (5). In addition to assessing the utility of dd-cfDNA in surveillance of allografts to detect rejection, the study also looked to delineate the correlation between dd-cfDNA and estimated glomerular filtration rate.

In a cohort of nearly 1,100 patients from over seven major transplant centers in the United States, dd-cfDNA measurements were done at regular intervals done as part of surveillance and for-cause in the setting of graft dysfunction to examine its “real world” application. Transplant kidney biopsies were performed as a part of the study in the setting of worsening creatinine, proteinuria and/or development of *de novo* donor specific antibody. One of the salient findings of the study was that a relative change in serial dd-cfDNA, in addition to an isolated absolute measurement, may signal allograft injury and dnDSA formation. An increase in dd-cDNA of ∼150% warrants consideration for closer monitoring and/or further investigation of potential graft injury.

Circulating Donor-Derived Cell-Free DNA in Blood for Diagnosing Acute Rejection in Kidney Transplant Recipients (DART) study demonstrated that a dd-cfDNA threshold of >1% aided in discerning presence of rejection (1). Data from the ADMIRAL further adds to understanding the interpretation of dd-cfDNA measurements-values < 0.5% were indicative of absence of allograft injury or presence of allograft quiescence (causes for injury included out-of-range tacrolimus level <4 ng/ml or >12 ng/ml, BK viremia, dnDSA-positive, urinary tract infection, proteinuria, allograft rejection, or recurrent focal segmental glomerulosclerosis). The investigators assessed dd-cfDNA as a marker of graft quiescence with paired biopsies <30 days after dd-cfDNA measurements. This shows that dd-cfDNA could bguide clinicians to avoid unnecessary investigations, including invasive procedures such as kidney transplant biopsies.

A decline in eGFR 1–3 years post kidney transplantation portends an increased risk of graft failure and death (6, 7). The ADMIRAL study demonstrated a correlation between elevated dd-cfDNA and eGFR decline during this period. Continually elevated dd-cfDNA (more than 1 result of >0.5%) was associated with doubling of risk of 25% decline in eGFR. This is the first study to show the correlation between dd-cfDNA and renal function decline, a measure that is pivotal in the real-world scenario. Persistent elevations in dd-cfDNA can signal not only the presence of possible ongoing allograft injury, but also forecast future decline of kidney allograft function.

While the ADMIRAL study expanded the repertoire of dd-cfDNA interpretation, it is important to address its limitations. Many dd-cfDNA and biopsy samples were not truly paired, with the investigators allowing for biopsies to be done within 30 days of dd-cfDNA measurements. It is possible that many early disease processes may have been missed or a new pathology may have arisen in the interim. This confounding cannot be accounted for, and future studies should endeavor to limit the duration elapsed between the dd-cfDNA measurement and subsequent kidney biopsy. There could also be observer bias since all biopsies were read locally and lacked centralized reporting. Given that this study was designed to assess the “real world” utility of dd-cfDNA, the investigators could have also assessed the correlation of dd-cfDNA with proteinuria. The presence of proteinuria is strongly associated with reduced graft survival (8) and since dd-cfDNA could now be a prognosticating tool, it would be important for future studies to examine the existence of a correlation between the two measurements. Lastly, in keeping with previous studies, dd-cfDNA appears to be more sensitive in detecting ABMR compared to TCMR (1, 5).

The Molecular Microscope Diagnostic System (MMDx) is a method of elucidating various pathologies on allograft biopsy sample by utilizing automatic genome-wide microarray measurements and interprets disease states by machine learning–derived classifiers and archetype scores (9). The Banff Molecular Diagnostics Work Group now recommends utilization of the Banff Human Organ Transplant gene expression panel consisting of 770 genes related to rejection, tolerance, and viral infections, and innate and adaptive immune responses (10). The correlation of disease effector gene transcripts, histology and dd-cfDNA has not been well defined until recently. The Trifecta study, an international prospective trial, assessed the relationship of dd-cfDNA done at the time of kidney allograft biopsy with gene transcriptomic signatures on the MMDx. In a cohort of 300 biopsies, the authors report a case representation similar to that of previous studies with 60% demonstrating no rejection, while the rest showing antibody mediated rejection (30%) and T-cell mediated (TCMR)/mixed rejection (10%). The top 20 gene transcripts (of 49,495 total probe sets) that have been previously shown to be highly associated with all types of rejection, correlated positively with dd-cfDNA. These gene transcripts mostly represented natural killer (NK) cells and those induced by interferon gamma.

The correlation of multigene measurement scores (transcript sets) with dd-cfDNA were strongest with ABMR and all-rejection scores, while being moderate with TCMR scores, and weak with recent parenchymal injury, dedifferentiation, and atrophy-fibrosis scores. The investigators performed a principal component analysis (PCA), in which the dd-cfDNA vector highly approximated the peritubular capillaritis molecular classifier vector in all three dimensions (all rejection, ABMR and early stage ABMR). Dd-cfDNA, therefore, correlated with an important component of the Banff classification used for diagnosis of ABMR-peritubular capillaritis.

Active rejection based on molecular measurements had the highest dd-cfDNA levels, while biopsies with no molecular or histologic evidence of rejection has the lowest values. Importantly, the molecular scores predicted dd-cfDNA ≥1.0% better than histologic scores. This finding adds further to accumulating evidence that histology, while regarded as the gold standard for diagnosing a vast array of allograft pathologies, may not correlate with extent of damage.

The Trifecta study findings of lower dd-cfDNA levels in TCMR in comparison to ABMR is in line with that of the DART study (1). The Trifecta investigators present an intriguing hypothesis to explain this phenomenon-the degree of dd-cfDNA released by TCMR reflects the activation state of the effector T cells in those TCMR biopsies. Previous archetypal analyses have established that TCMR has two phenotypes varying in molecular activity- TCMR1 (intense TCMR, sometimes mixed with ABMR) and TCMR2 (less active TCMR). While the TCMR1 phenotype has more intense interferon gamma expression, it can also have some ABMR features, in comparison to TCMR2. Therefore, explaining release of dd-cfDNA, which has strongly correlated with ABMR and interferon gamma activity in this study. However, TCMR with lower dd-cfDNA levels may have T-cells with attenuated activity as corollary of immunosuppression or exhaustion. It is also important to note that all biopsies included in this study were “for cause” and no subclinical features of rejection were investigated. Therefore, it is difficult to assess the correlation of dd-cfDNA with incipient subclinical rejection. Morever, some cases had high levels of dd-cfDNA with absence of biopsy proven rejection.

Histologic lesions of borderline and TCMR 1A can exhibit considerable overlap, with clinical relevance of either lesions and optimal treatment continues to debated (11). However, it is being increasingly recognized that borderline TCMR portends to inferior graft outcomes even in the event of subsequent resolution of inflammatory infiltrates (12). An objective measure that could aid in discerning actual presence of tissue damage in the presence of these lesions could augment the Banff diagnostic categories.

Previous studies elucidating the use of dd-cfDNA in kidney allograft rejection demonstrated that a proportion of patients with TCMR 1A did not have elevated levels (1, 13). It could be argued that this subset of patients may not actually have a true rejection episode with the infiltrate devoid of any deleterious effects. A multicenter study assessed if elevated dd-cfDNA was associated with adverse outcomes in patients with borderline and TCMR 1A rejection (14). Over a 3-year period, in the cohort with elevated dd-cfDNA (>0.5%) the estimated glomerular filtration rate declined by 8.5% (vs. 0% in those with low dd-cfDNA <0.5%), *de novo* donor specific antibody (dnDSA) was seen in 40% (vs. 2.7%) and future or persistent rejection occurred in 22% (vs. 0%). This study demonstrates that dd-cfDNA could be used to detect early rejection and aid in discerning which lesions are actually associated with injury, which in turn, are associated future adverse consequences. In addition, authors of this study have put forward a recommendation that a threshold of 0.5% be considered for indicating damage/rejection, with interpretation of this test be as a continuous variable.

## The *de novo* DSA Link and Measurement Predicament

The generation of dnDSA is associated with adverse consequences, including development of antibody mediated rejection and eventual graft loss. Patients with dnDSA have a significant reduction in 10-year graft survival in comparison to those who do not (57% vs. 96%) (15). As with early rejection entities borderline and TCMR 1A, data for risk stratification by type of dnDSA is lacking and there is no established agreement on treatment once dnDSA has been detected. There is emerging evidence that dd-cfDNA may be a potent stimulator of immune mediated inflammation (16). A retrospective cohort study of the Circulating Donor-Derived Cell-Free DNA in Blood for Diagnosing Acute Rejection in Kidney Transplant Recipients (DART) assessed the association of dnDSA and dd-cfDNA (17). Levels of dd-cfDNA were higher in patients with dnDSA compared to those with none. Elevated dd-cfDNA (>1%) in the first-year post transplant year is associated with eGFR decline of >25% in the following year. It is important to note that patients with rejection were excluded in this cohort and the finding of higher dd-cfDNA is likely reflective of ongoing subclinical allograft injury, with demonstration of eventual decline in eGFR. Utilization of dd-cfDNA in concurrence with dnDSA may aid in discerning pathogenic from non-pathogenic antibodies and identifying patients at high risk for future allograft dysfunction, who may benefit from augmentation of immunosuppression.

The ADMIRAL study provided further granularity to relationship between dd-cfDNA and dnDSA (5). Dd-cfDNA levels >0.5% was associated with a 3-fold higher risk of dnDSA production in the future, with persistent elevation of dd-cfDNA in all patients with detectable dnDSA. Additionally, every 1% increase in dd-cfDNA levels was associated with a 20% increase in risk of dnDSA formation, and a median increase of ∼120% in dd-cfDNA from previous values occurred at a median of 91 days prior to development of dnDSA.

Another study investigated the diagnostic value of dd-cfDNA when added to DSA in detecting ABMR in two independent cohorts of kidney transplant patients (one cohort with subclinical cases identified with DSA testing >180 days post transplantation and the other with indication biopsies >1 month post transplantation) (18). The addition Dd-cfDNA to DSA or vice-versa significantly improved the diagnostic yield in identifying ABMR in the first cohort. However, the combination of DSA and dd-cfDNA did not translate into a similar diagnostic value given disparate number of biopsy proven diagnosis in the indication biopsy cohort, which included TCMR, glomerulonephritis and BK associated nephropathy. While this study strengthens the diagnostic axis of dd-cfDNA, DSA and AMBR, the diagnostic accuracy of dd-cfDNA in identifying other pathologies remains suboptimal.

Given dd-cfDNA is calculated as percentage of the total circulating DNA (donor derived and recipient derived cell free DNA), any change in background cell free DNA may result in a false result. High body mass index (BMI) and increasing age may result in higher background cell free DNA given association with increased inflammation and escalated cell senescence respectively (19, 20). A study examined this plausible effect of BMI and age on dd-cfDNA demonstrating a significant negative correlation between increasing BMI and baseline dd-cfDNA levels, with no influence of age on the biomarker (21). This, albeit, being a small study, highlights the need for further studies to assess the influence of BMI on dd-cfDNA and if levels need to adjusted based on body habitus. Clinicians should be mindful of possible falsely low levels in the setting of high BMI, which may in turn, lead to missing evolving rejection.

## The Pandemic Angle

The COVID-19 pandemic necessitated dramatic changes in delivery of healthcare. From a transplant perspective, measures to reduce exposure to the virus and augmenting vaccine response have been the most essential initiatives to mitigate the viral infection in the vulnerable transplant population. Telemedicine and remote home phlebotomy were employed as methods to minimize healthcare associated exposure the virus.

Two studies demonstrated that dd-cfDNA drawn via remote home phlebotomy could be utilized for surveillance of allografts (22, 23). This aided in identifying patients at risk of rejection and subsequent triage for allograft biopsies. These studies did not identify if this reduced the need or could be a replacement for protocol biopsies, however, they do represent a potential blueprint for allograft monitoring for subsequent waves of COVID-19 and future pandemics.

## Beyond Conventional Rejection

**FIGURE 1:**
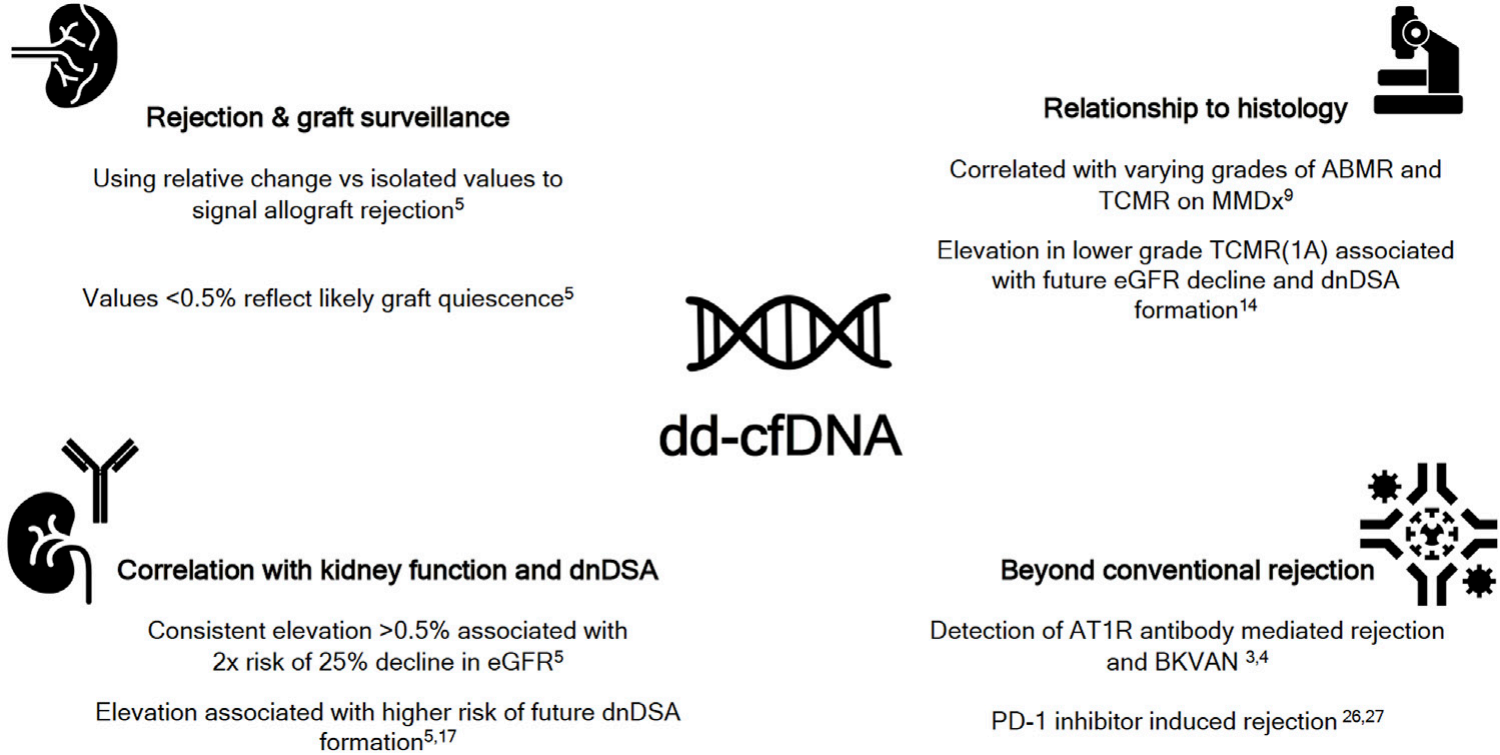
The expanding paradigm of donor derived cell free DNA (dd-cfDNA). ABMR-antibody mediated rejection; AT1R, angiotensin 1 receptor; BKVAN, BK virus associated nephropathy; dnDSA, *de novo DSA*; eGFR, estimated glomerular filtration rate; MMDx, molecular microscope; PD-1, programmed cell death-1; TCMR, T cell mediated rejection.

The utilization of dd-cfDNA has been extensively validated in TCMR and HLA antibody induced antibody mediated rejection (ABMR). It is now being employed beyond these conventional indications (Figure 1):

(1) Angiotensin-1 receptor (AT1R) antibody mediated rejection: the presence of AT1R antibodies has been demonstrated to be independently associated with high risk for development of ABMR and decreased long term graft survival (24). However, these antibodies can be present prior to transplantation, its levels cannot predict presence of rejection and a proportion of patients with the antibodies do not eventually develop rejection (25). A multicenter study involving with patients with biopsy proven ABMR and pre-existing positive AT1R antibodies, showed that dd-cfDNA correlated well with Banff components of rejection (3). Therefore, dd-cfDNA could be utilized to for surveillance and detection of incipient rejection in this setting.
(2) Anti-programmed cell death-1 (PD-1) inhibitor induced rejection: Immune check point inhibitors are being increasingly used to treated numerous cancers. In addition to being associated with multi-systemic adverse effects, allograft rejection can be a devastating consequence of these agents (26-28). Two case reports demonstrated the use of dd-cfDNA for monitoring for rejection while successfully continuing PD-1 inhibitor therapy. Larger studies are required to validate these preliminary reports (27, 28).
(3) Distinguishing BK virus associated nephropathy (BKVAN) from BK viremia (BKV): it can be challenging to discern progression of BKV to BKVAN- especially with reliance on the often debated cut off viral load of >10,000 copies/mL and eventual allograft biopsy, which itself is associated with discordant reads (29, 30). A retrospective analysis of the DART study demonstrated that dd-cfDNA could distinguish BKV from BKVAN, and that levels of dd-cfDNA correlated with BK viral loads (4).

## Conclusion

As dd-cfDNA continues to integrate into surveillance regimes of kidney allografts, some aspects continue to remain unanswered. There is yet to be a defined frequency of dd-cfDNA testing substantiated by a robust clinical study (31). The Kidney Allograft Outcomes Registry (KOAR) study (NCT033226076) will look to assess this aspect with planned dd-cfDNA testing at various pre-defined intervals along with planned 12-month allograft biopsies-this will also aid in ascertaining if dd-cfDNA could reduce the need for protocol biopsies. This biomarker is predominantly beneficial in detecting alloimmune damage, however, has no utility in identifying non-immune causes such as acute tubular injury. Further nuance is definitely required to determine the optimal threshold of dd-cfDNA to proceed with allograft biopsy and identify patients that can be safely monitored since high levels can be present in the absence of rejection. Larger studies are also required to elucidate whether absolute graft derived cfDNA or fractionated measurements are more accurate in detection of rejection, along with appropriate context of their application.
